# A Rare Case of Severe Autoimmune Hypothyroidism Presenting With Massive Pericardial Effusion, Mimicking Malignancy, and Complicated by Seizures

**DOI:** 10.7759/cureus.97288

**Published:** 2025-11-19

**Authors:** Mahmudul Hasan Nahid, Arooba Rubbani, Reema Munshi, Muhammad Faisal Saleem

**Affiliations:** 1 Acute Internal Medicine, University Hospitals Leicester, Leicester, GBR; 2 Internal Medicine, Shaheed Ziaur Rahman Medical College, Bogura, BGD; 3 Medicine, University Hospitals of Leicester NHS trust, Leicester, GBR; 4 Internal Medicine, University Hospitals of Leicester, Leicester, GBR; 5 Internal Medicine, University Hospital of Morecambe Bay NHS trust, Lancaster, GBR

**Keywords:** autoimmune hypothyroidism, ca-125, cardiac tamponade, endocrine emergency, hashimoto’s thyroiditis, impending pericardial effusion, myxoedema, myxoedema heart

## Abstract

Severe hypothyroidism is a recognised but uncommon cause of large serous effusions, and massive pericardial involvement with haemodynamic compromise remains particularly rare. We describe a 39-year-old woman presenting with progressive dyspnoea, peripheral oedema, and abdominal distension. Imaging revealed a large circumferential pericardial effusion measuring 5.8 cm with tamponade physiology, accompanied by bilateral pleural effusions and ascites. Serum CA-125 was markedly elevated, prompting concurrent oncologic and endocrine evaluation. She underwent emergency pericardiocentesis for cardiac tamponade before the underlying cause was known, which yielded serous, non-malignant fluid with benign cytology. Cardiac, hepatic, and renal investigations were unremarkable, and subsequent endocrine testing confirmed severe primary hypothyroidism (thyroid-stimulating hormone (TSH) >150 mIU/L, undetectable free thyroxine) with positive thyroid peroxidase antibodies, consistent with autoimmune Hashimoto’s thyroiditis. The patient was commenced on combined levothyroxine and liothyronine therapy, after which she developed brief self-limiting generalised seizures attributed to mild hyponatraemia during metabolic recovery. With gradual thyroid hormone replacement, her cardiovascular function stabilised and all serous effusions resolved completely. This case highlights a rare presentation of severe autoimmune hypothyroidism with massive pericardial effusion causing tamponade, multi-serosal involvement, and transient metabolic seizures, demonstrating its ability to mimic malignant or systemic disease while remaining fully reversible with timely endocrine therapy.

## Introduction

Hypothyroidism is a prevalent endocrine disorder with a wide range of systemic manifestations. While fatigue, cold intolerance, and weight gain are well recognised, their cardiovascular and serosal effects are less frequently appreciated. Pericardial effusion is a known but often under-recognised manifestation of untreated hypothyroidism, and clinically significant fluid accumulation can develop insidiously even in the absence of classical symptoms. Simultaneous involvement of multiple serous compartments, however, remains uncommon [1,2]. The underlying pathophysiology involves increased capillary permeability, reduced lymphatic drainage, and the gradual accumulation of protein-rich fluid within serous spaces, typically without early haemodynamic compromise [2].

When a patient presents with large serous effusions involving the pericardium, pleura, or peritoneum, the diagnostic evaluation usually begins with exclusion of cardiac, hepatic, and renal causes. Once these are ruled out, elevated tumour markers such as CA-125 may prompt investigation for malignancy; however, several studies have shown that CA-125 can also rise in benign inflammatory or endocrine-related effusions, reflecting nonspecific mesothelial activation rather than neoplastic disease [3,4]. This overlap complicates early diagnostic interpretation, especially in younger patients without clear comorbidities, and emphasises the need for concurrent systemic, oncologic, and endocrine assessment.

In severe hypothyroidism, central nervous system involvement may occur, leading to reversible neurocognitive changes or, rarely, seizure activity. Proposed mechanisms include hyponatraemia, reduced cerebral perfusion, and altered neuronal excitability associated with profound thyroid hormone deficiency [5,6].

We report a diagnostically challenging case of a previously healthy 39-year-old woman who presented with progressive multi-serosal effusions and elevated tumour markers. Following exclusion of cardiac, hepatic, and renal pathology, concurrent oncologic and endocrine assessment revealed severe autoimmune hypothyroidism. Her course was further complicated by transient seizure activity during early thyroid hormone replacement. This case highlights the protean nature of hypothyroidism and underscores the importance of maintaining clinical suspicion for endocrine causes in patients with unexplained effusions.

## Case presentation

A 39-year-old woman with no prior medical history was referred by her general practitioner with a two-week history of progressive lower-limb swelling, shortness of breath, and fatigue. She also reported abdominal distension and reduced exercise tolerance. There was no history of chest pain, palpitations, weight loss, or flank pain. She was not taking any regular medication and denied alcohol or illicit drug use.

On admission, she appeared drowsy and peripherally cool. Her temperature was 35.1°C, blood pressure 86/54 mmHg, heart rate 54 beats per minute, and oxygen saturation 90 % on room air, improving with 4 L of oxygen. Cardiovascular examination revealed muffled heart sounds and raised jugular venous pressure. Respiratory examination showed decreased air entry bilaterally at the lung bases. Bilateral pitting oedema was present up to the knees, and the abdomen was distended but non-tender with shifting dullness, consistent with ascites.

**Figure 1:**
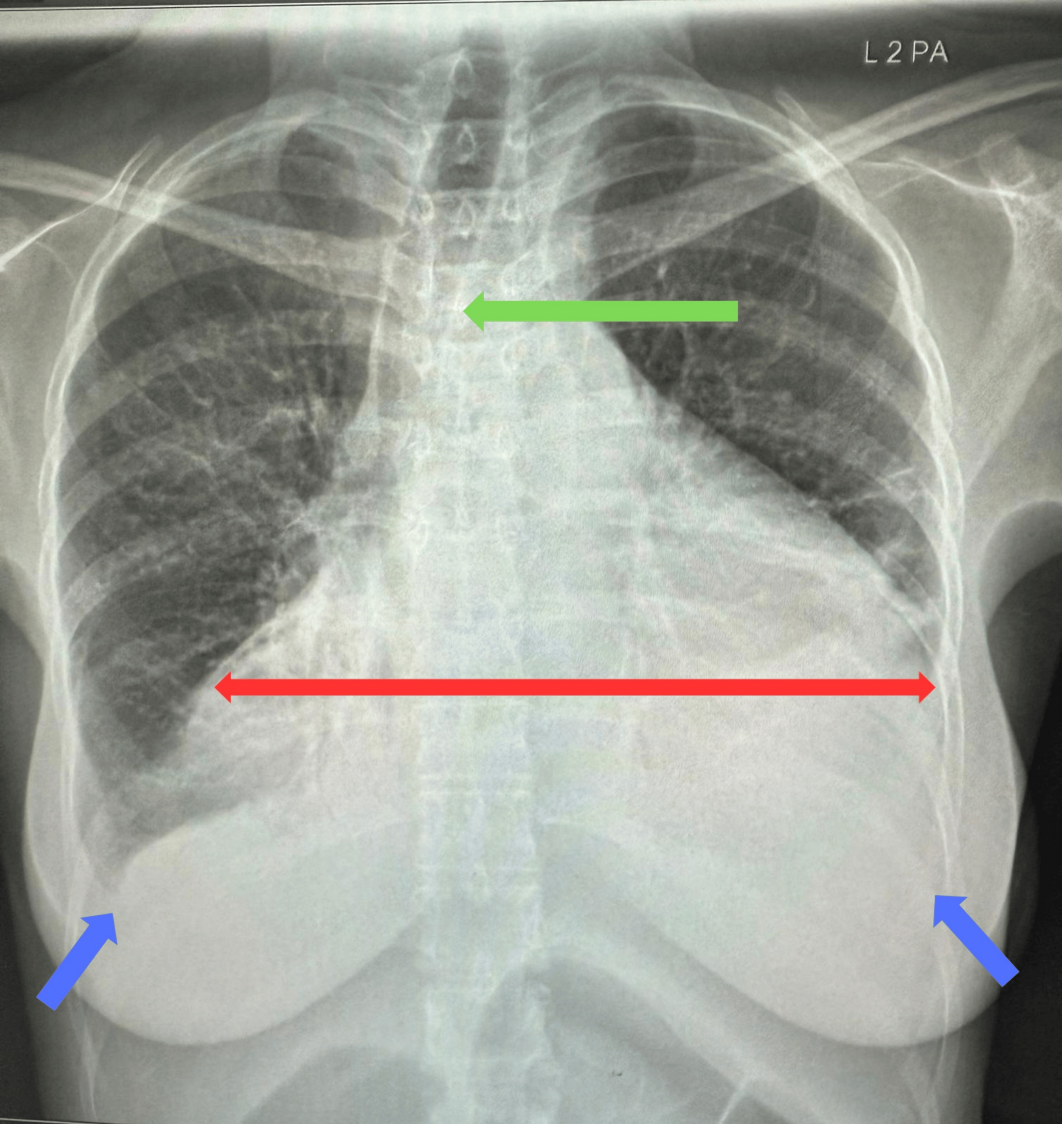
Chest X-ray showing cardiomegaly (Red), bilateral blunt costophrenic angles (Blue), and deviated trachea (Green).

A chest X-ray demonstrated marked cardiomegaly with bilateral costophrenic angle blunting, suggestive of pleural effusion (Figure 1).

**Figure 2:**
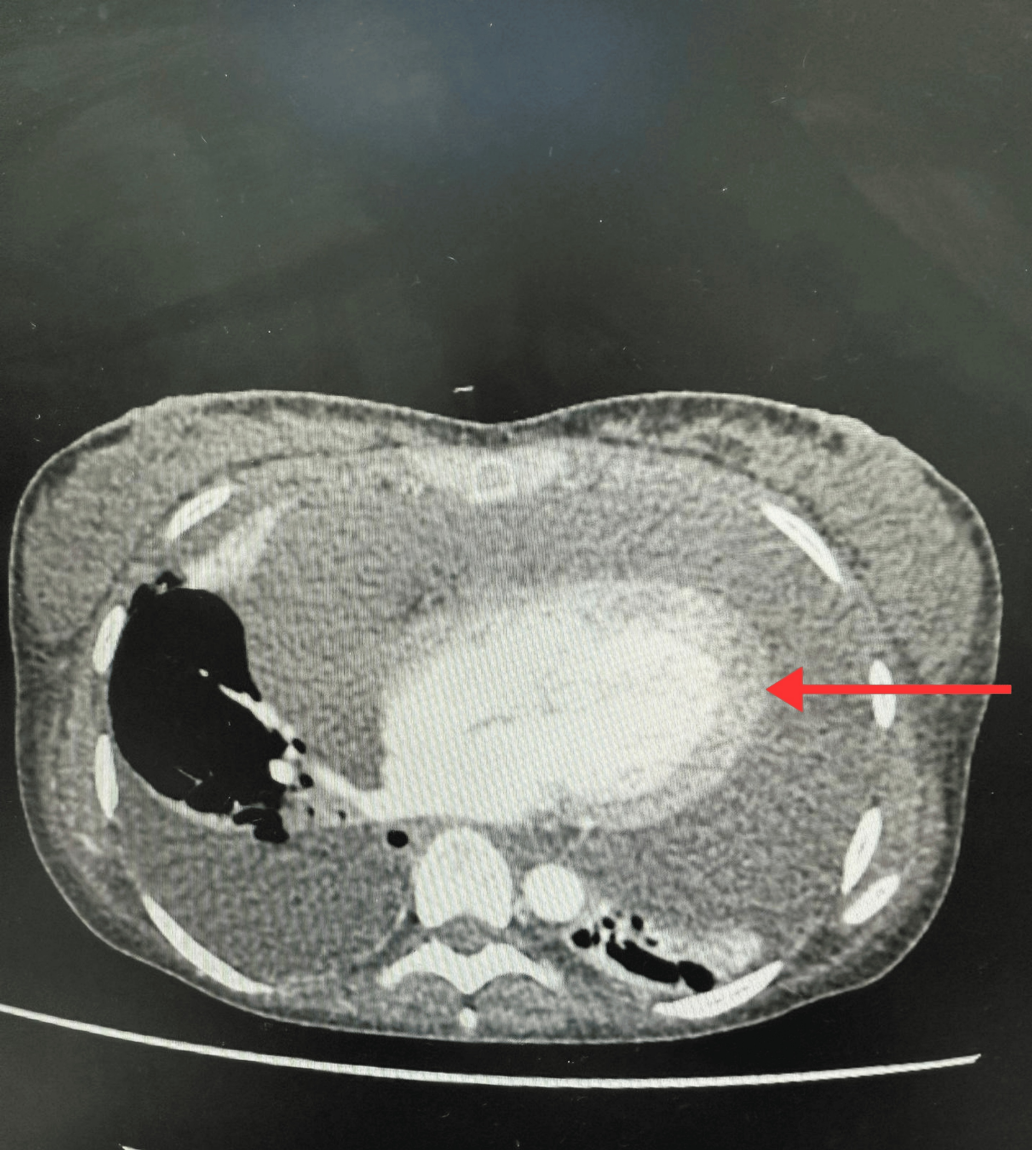
A cross-sectional image of CT chest-abdo-pelvis showing marked pericardial effusion (Red arrow).

A contrast-enhanced CT scan of the chest, abdomen, and pelvis revealed a large circumferential pericardial effusion measuring up to 54 mm inferiorly, with echocardiographic features of hemodynamic compromise, alongside large-volume ascites and bilateral pleural effusions (Figure 2).

The liver, kidneys, and other abdominal organs appeared normal.

An urgent transthoracic echocardiogram confirmed a large global pericardial effusion (maximum diameter 5.8 cm) with right ventricular free wall collapse during diastole and a “swinging heart” appearance, indicating significant hemodynamic compromise. The left ventricular ejection fraction was preserved at 50 %. The patient underwent emergency pericardiocentesis, which drained a large volume of serous fluid and resulted in prompt haemodynamic improvement. A summary of echocardiographic findings before and after pericardiocentesis is presented in Table 1, demonstrating complete resolution of tamponade physiology and restoration of haemodynamic stability.

**Table 1:** Summary of key echocardiographic findings before and after pericardiocentesis, demonstrating marked resolution of pericardial effusion and haemodynamic recovery. RV: right ventricle; LV: left ventricle; RA: right atrium

Parameter	Before Pericardiocentesis	After Pericardiocentesis
Pericardial effusion	Very large circumferential effusion (max 5.8 cm) with RV diastolic collapse and 'swinging heart' motion suggesting tamponade physiology.	Complete resolution of pericardial effusion; normal cardiac motion restored.
Left ventricular function	Difficult to assess due to large effusion; LV non-dilated, function uncertain.	Low-normal systolic function, EF ≈ 50%.
Right ventricular function	RV free wall collapse in diastole; systolic function indeterminate.	Normal size and systolic function.
Inferior vena cava	Dilated and fixed with possible echogenic thrombus.	Normal calibre and collapsibility (no thrombus reported).
Atria	Non-dilated; RA poorly visualised.	Non-dilated bilaterally.
Valvular findings	No significant valvular disease.	No significant valvular disease.
Overall impression	Features of cardiac tamponade due to severe pericardial effusion.	Complete resolution of tamponade physiology; haemodynamic stability restored.

Initial laboratory investigations showed normal renal and liver function, normal serum albumin, and mild proteinuria (1 +) with a slightly elevated protein-creatinine ratio. The coagulation profile was normal. Brain natriuretic peptide (BNP) levels were within the normal range, making heart failure less likely. A full blood count revealed microcytic anaemia, which was under investigation to rule out iron deficiency anaemia.

A summary of baseline laboratory results is presented in Table 2.

**Table 2:** Routine investigation results with their reference ranges eGFR: estimated glomerular filtration rate; CKD-EPI: chronic kidney disease-Epidemiology Collaboration; ALT: alanine transaminase; INR: international normalized ratio; APTT: activated partial thromboplastin time

Test	Value	Reference Range
White cell count	7.9 ×10⁹/L	4.0 – 11.0
Red blood count	3.64 ×10¹²/L	3.90 – 5.60
Haemoglobin	83 g/L	115 – 165
Haematocrit	0.266 L/L	0.370 – 0.470
Mean cell volume (MCV)	73 fL	80 – 100
Mean cell haemoglobin (MCH)	22.7 pg	27 – 32
Platelet count	183 ×10⁹/L	140 – 400
Neutrophil count	6.73 ×10⁹/L	2.0 – 7.5
Lymphocyte count	0.71 ×10⁹/L	1.0 – 4.0
Monocyte count	0.36 ×10⁹/L	0.2 – 0.8
Eosinophil count	0.02 ×10⁹/L	0.04 – 0.40
Basophil count	0.02 ×10⁹/L	0.00 – 0.20
C-reactive protein (CRP)	21 mg/L	0 – 10
ALT	32 U/L	10 – 49
Total bilirubin	10 µmol/L	0 – 21
INR	0.9	-
Prothrombin time	12.7 secs	12.0 – 15.0
APTT	31.1 secs	26.1 – 36.6
APTT ratio	1	-
Total protein	66 g/L	57 – 82
Albumin	38 g/L	35 – 50
Total calcium	2.16 mmol/L	2.12 – 2.51
Adjusted calcium	2.2 mmol/L	2.12 – 2.51
Inorganic phosphate	1.07 mmol/L	0.80 – 1.50
Alkaline phosphatase (ALP)	157 U/L	30 – 130
Na	128 mmol/L	133-146
K	4.4 mmol/L	3.5-5.3
urea	7.5 mmol/L	2.5-7.8
creatinine	82 umol/L	60-120
eGFR (CKD-EPI)	79 mL/min/1.73 m²	>90

Case-specific and serial trends in cardiac and biochemical parameters during admission are shown in Table 3.

**Table 3:** Case-specific investigation results with their reference ranges (if available) TSH: thyroid-stimulating hormone; ENA: extractable nuclear antigen; BNP: brain natriuretic peptide; CEA: carcinoembryonic antigen; ENA: extractable nuclear antigen

Test	Value	Reference Range
Autoimmune profile		
Thyroid peroxidase antibody (TPO)	68 IU/mL	<25 Neg, 25–35 Eq, >35 Pos
Anti-TSH receptor antibody (TSHR)	Negative	-
ANCA (immunofluorescence)	Negative	
MPO-ANCA	<1.0 IU/mL	<3.5 Neg, >= Pos
PR3-ANCA	<0.6 IU/mL	0-5
dsDNA antibody	18.8 IU/mL	25-30
ENA antibody screen	Negative	
Endocrine Profile		
Free T3 (FT3)	<0.3 pmol/L	3.5 – 6.5
Free T4 (FT4)	<2.0 pmol/L	9.7 – 24.7
Thyroid-stimulating hormone (TSH)	>150 mIU/L	0.55 – 4.78
Luteinising hormone (LH)	<0.5 IU/L	Follicular 2.5–12.5
Follicle-stimulating hormone (FSH)	3.6 IU/L	Follicular 3.5–12.5
Prolactin (PRL)	583 mIU/L	59 – 619
Serum vitamin B12	847 ng/L	211 – 911
Sex hormone binding globulin (SHBG)	70 nmol/L	17.7 – 138.3
Cortisol	989 nmol/L	-
Urine Studies		
Random protein	0.12 g/L	0.01-0.14
Random creatinine	3.1 mmol/L	
Protein/creatinine ratio	38.7 mg/mmol	0-30
Tumour Markers		
CA 125	303 kU/L	0-35
CEA	4.4 ug/L	0-2.5
Cardiac marker		
NT-pro BNP	(245 initially) 9333 pg/mL	100-400

Given the presence of multi-serosal effusions (pericardial, pleural, and ascitic) and an elevated CA-125 level (291 U/mL) with mildly raised CEA, a malignancy workup was initiated. An MRI of the abdomen and pelvis revealed no evidence of ovarian, gastrointestinal, or peritoneal malignancy. Concurrently, endocrine testing was arranged due to persistent bradycardia, hypothermia, and facial puffiness. Thyroid function testing revealed severe primary hypothyroidism with TSH > 150 mIU/L and undetectable free T4. Thyroid peroxidase antibodies were strongly positive, confirming autoimmune (Hashimoto’s) thyroiditis as the underlying cause. Autoimmune screening demonstrated positive antinuclear antibodies (ANA) but was negative for anti-dsDNA and ENA antibodies.

The patient was commenced on thyroid hormone replacement therapy with levothyroxine (75 µg daily) and liothyronine (10 µg daily), alongside cautious fluid management and gradual rewarming.

**Figure 3:**
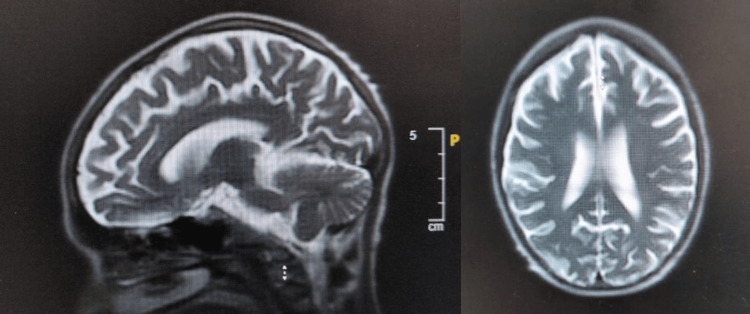
MRI Brain (T2-weighted sequences) Left: Sagittal view showing diffuse cerebral and cerebellar volume loss disproportionate to age. Right: Axial view demonstrating global sulcal widening and mild ventricular prominence without a focal lesion.

Following initiation of thyroid replacement, she experienced three self-limiting episodes of generalised tonic-clonic seizures within 24 hours, each lasting under one minute and followed by transient post-ictal confusion. Neurology was consulted promptly. Neurological examination revealed no focal deficits, and investigations were performed to evaluate potential metabolic or structural causes. MRI of the head demonstrated diffuse cerebral and cerebellar involutional changes disproportionate to age, but no acute pathology. Neurology interpreted the volume loss as nonspecific and not clinically actionable, given the absence of focal findings and complete neurological recovery after metabolic stabilisation. Representative MRI images are shown in Figure 3. Electroencephalography (EEG) was normal, and lumbar puncture (LP) revealed clear cerebrospinal fluid with normal cell counts, protein, and glucose. Autoimmune and neuronal antibody panels were sent and remained pending at the time of discharge.

Given the context of severe hypothyroidism and mild hyponatraemia, the seizures were attributed to metabolic instability rather than autoimmune encephalopathy. She was commenced on a regular antiepileptic regimen with levetiracetam for seizure prophylaxis, with good response and no recurrence during admission.

**Figure 4:**
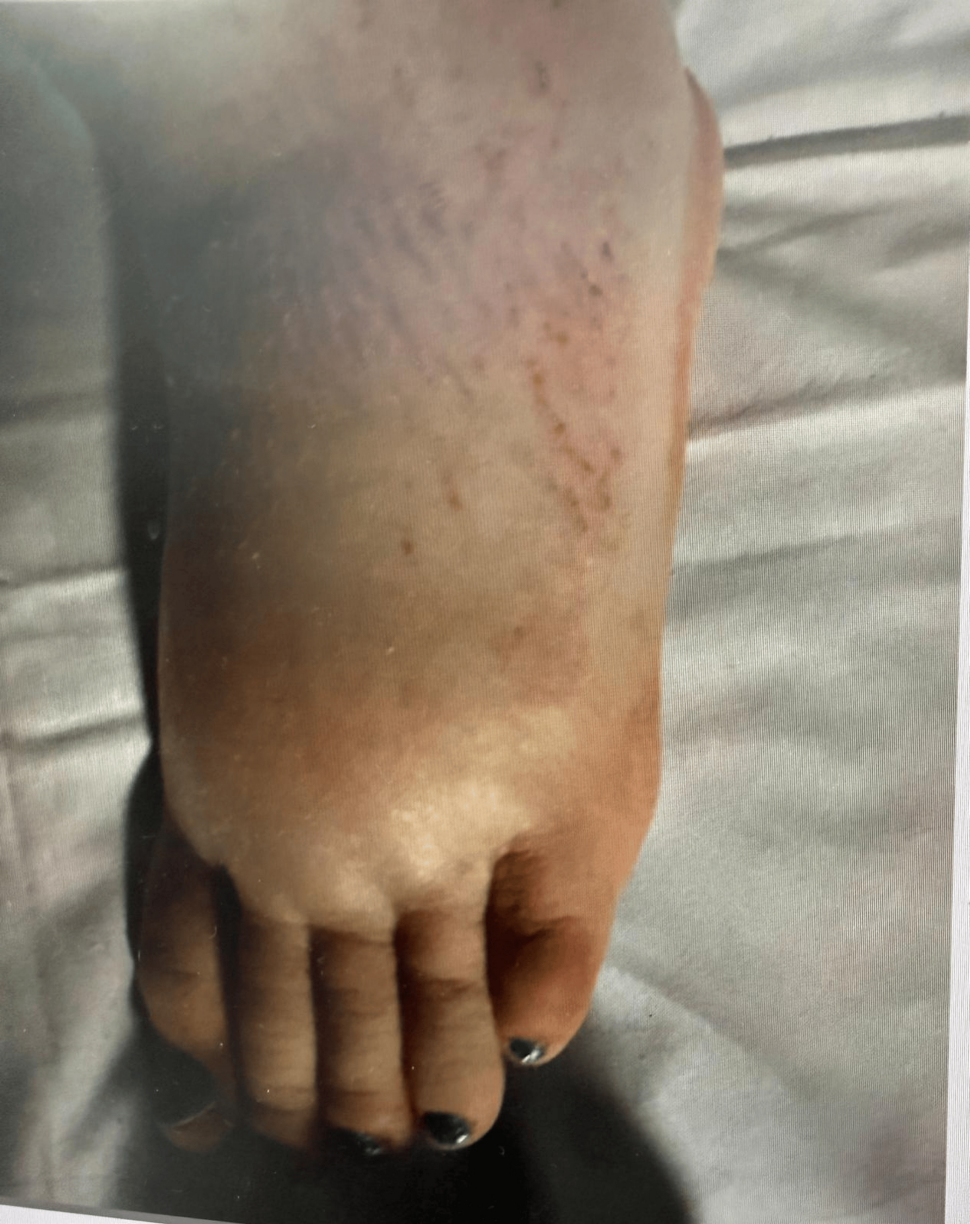
Initial presentation of pitting oedema

**Figure 5:**
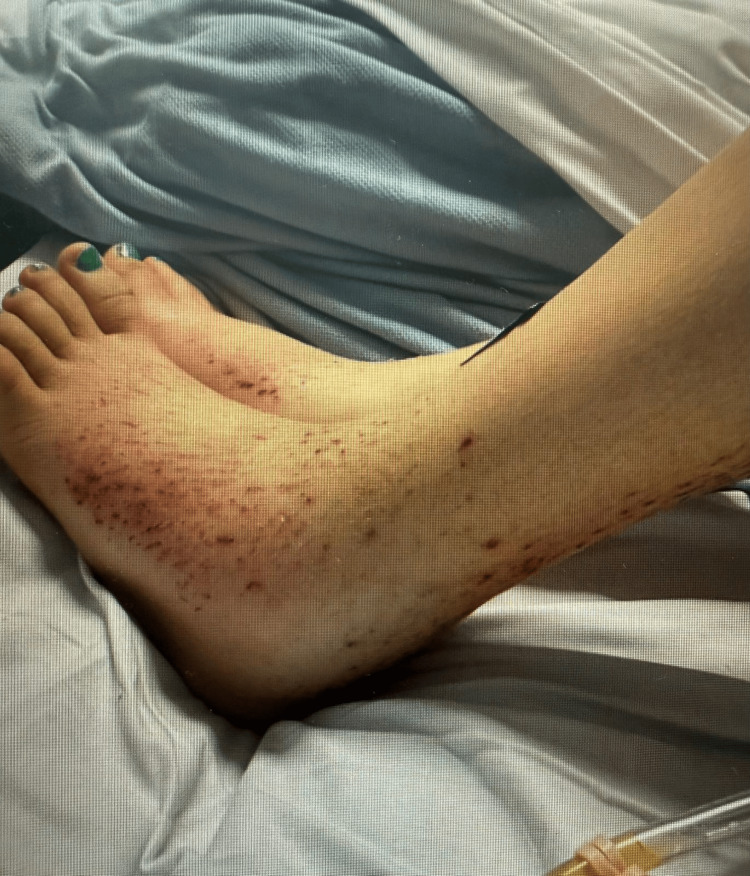
Improvement of pedal oedema after treatment

Over the subsequent days, her condition improved steadily. Blood pressure and temperature normalised, oxygen supplementation was discontinued, and there was marked resolution of facial and lower-limb oedema. A repeat echocardiogram confirmed sustained resolution of the pericardial effusion. Clinical photographs documenting the resolution of bilateral lower-limb oedema following thyroid hormone replacement are shown in Figures 4-5.

By discharge, she was alert, oriented, and haemodynamically stable with normal urine output. She was scheduled for follow-up with endocrinology and cardiology for ongoing thyroid hormone titration, thyroid function monitoring, and interval echocardiographic review.

## Discussion

This patient’s presentation with progressive dyspnoea, peripheral oedema, and large multi-serosal effusions prompted an initial search for a systemic cause of fluid overload. In clinical practice, cardiac failure, hepatic cirrhosis, and renal disease are recognised as the predominant non-malignant causes of pleural and peritoneal effusions [7]. Accordingly, these were investigated first. Normal liver and renal indices, preserved left-ventricular systolic function, and the absence of nephrotic-range protein loss made cirrhosis and nephrotic syndrome unlikely, findings consistent with published diagnostic approaches to transudative effusions [7].

Because of the patient’s sex and the presence of elevated CA-125, malignancy was concurrently considered. Elevated CA-125 levels often prompt investigation for ovarian or peritoneal malignancy, yet published evidence has shown that this glycoprotein is a nonspecific mesothelial marker that can also rise in benign inflammatory or endocrine-related effusions. Comparative studies have demonstrated that CA-125 levels in benign peritoneal irritation or non-malignant ascites may overlap with levels seen in ovarian cancer [8,9]. In this case, cytological examination of the pericardial fluid revealed a histiocytic effusion without malignant cells - a finding consistent with previous reports of inflammatory, autoimmune, or hypothyroid-related pericardial effusions [10]. Given these results, an extended autoimmune screen was performed to consider connective-tissue disease, including lupus serositis. Although ANA was positive, the absence of anti-double-stranded DNA and extractable nuclear antigen antibodies made systemic lupus erythematosus and related autoimmune conditions unlikely, further supporting an endocrine rather than rheumatologic aetiology.

During this same period, additional features emerged that suggested an endocrine cause. The patient developed bradycardia, hypothermia, and facial puffiness, clinical hallmarks of severe hypothyroidism and the “myxoedema heart” phenotype, in which profound thyroid hormone deficiency depresses cardiac output and sympathetic drive while promoting serous effusion formation [1,2]. These patients may appear haemodynamically compromised but remain bradycardic rather than tachycardic, a paradoxical presentation characteristic of hypothyroid physiology [2]. Thyroid function testing confirmed severe primary hypothyroidism (TSH > 150 mIU/L, undetectable free T4), and high-titre thyroid peroxidase antibodies established autoimmune Hashimoto’s thyroiditis as the underlying cause. Such antibody positivity carries high sensitivity and specificity for autoimmune thyroid disease [11], unifying the cardiovascular, serosal, and systemic findings.

Once autoimmune hypothyroidism was confirmed, the overall clinical picture in this patient was most consistent with a decompensated hypothyroid state, or myxoedema. Severe thyroid hormone deficiency promotes interstitial accumulation of glycosaminoglycans, increases capillary permeability, and impairs lymphatic drainage, allowing protein-rich fluid to collect in the pericardial, pleural, and peritoneal cavities despite normal organ function [2,12,13]. This mechanism explains the large effusions seen in this patient without evidence of heart failure, cirrhosis, or nephrotic syndrome. The associated hypotension, cool extremities, and marked bradycardia likewise reflect reduced cardiac output and blunted adrenergic tone [12,4]. The CT and follow-up imaging (Figures 1-2) and the visual resolution of oedema (Figure 3) demonstrate the reversibility of these endocrine-mediated effusions once hormone therapy was initiated [2,12,13,4].

Although the initial B-type natriuretic peptide was low, the subsequent marked rise in NT-proBNP (from 245 pg/mL to 9,333 pg/mL) coincided with large pericardial effusion and haemodynamic compromise. This supports the concept of functional low-output cardiac strain in severe hypothyroidism (“myxoedema heart”) rather than primary structural heart failure. Recent clinical data demonstrate that thyroid hormone deficiency can increase ventricular wall stress and upregulate natriuretic peptide secretion, producing high NT-proBNP concentrations even in the absence of intrinsic cardiomyopathy [14,15]. Once euthyroidism is restored, these values typically normalise, reflecting recovery of cardiac contractility and vascular tone [4,15].

Neurological involvement in severe hypothyroidism is rare but documented. Myxoedema coma and advanced hypothyroid states have been associated with reversible encephalopathy, confusion, and occasionally generalised tonic-clonic seizures [5,16]. Mechanistically, hypothyroidism may reduce cerebral blood flow and glucose utilisation, lowering neuronal energy reserve [16], and it can also promote water retention and dilutional hyponatraemia through impaired free-water clearance. Hyponatraemia is a recognised precipitant of seizures in endocrine emergencies [5,16], and the serum sodium of 128 mmol/L in this case likely contributed to the transient events. Experimental work further shows that thyroid hormone deficiency alters neuronal excitability and lowers seizure threshold even in structurally normal brains [6]. In this patient, neuroimaging and cerebrospinal fluid studies were normal, and symptoms resolved completely after correction of thyroid hormone and sodium levels, consistent with a metabolic rather than structural mechanism.

Hashimoto’s encephalopathy was considered but excluded, as that syndrome typically presents with cognitive decline, psychosis, or stroke-like deficits with EEG or CSF abnormalities [17]. None of these were observed here, supporting transient metabolic seizure activity rather than autoimmune encephalopathy.

Following the identification of autoimmune hypothyroidism, thyroid hormone replacement became the central therapeutic measure. Combined levothyroxine (T4) and liothyronine (T3) therapy was selected to provide gradual metabolic correction with early physiological stabilisation. Restoration of thyroid tissue hormone enhances cardiac contractility, increases β-adrenergic receptor density, and improves systemic perfusion while promoting renal sodium and water excretion [4]. These mechanisms explain the patient’s cardiovascular recovery, rising blood pressure, improved temperature regulation, and steady resolution of effusions and oedema (Figures 2-3).

During the early treatment phase, she experienced brief generalised tonic-clonic seizures, attributed to transient metabolic instability. To prevent recurrence, short-term anticonvulsant therapy was prescribed. This approach is supported by evidence that sodium channel-modulating antiepileptics reduce neuronal hyperexcitability in acute symptomatic seizure contexts until metabolic homeostasis is restored [10]. No further seizures occurred after metabolic and endocrine stabilisation.

This case, therefore, underscores several learning points. First, severe hypothyroidism can present with multi-serosal/serous cavity effusions, reversible heart failure, and metabolic seizures, all of which are reversible with timely thyroid hormone replacement. Second, thyroid function should be tested early in patients with unexplained effusions, especially when cytology is non-malignant and tumour markers such as CA-125 are nonspecific [8,9]. Third, integration of endocrinology, cardiology, and neurology expertise is essential for safe correction of metabolic derangements and to prevent transient complications during treatment. The patient’s rapid improvement and radiological recovery highlight the extent to which advanced hypothyroid decompensation remains fully treatable when recognised promptly.

## Conclusions

This case demonstrates the profound yet reversible systemic consequences of severe autoimmune hypothyroidism. The combination of multi-serosal effusions, haemodynamic compromise, and transient neurological disturbance can closely mimic cardiac, hepatic, or malignant disease, leading to potential diagnostic delay. Recognition of the endocrine basis is crucial, as timely thyroid hormone replacement restores metabolic homeostasis and reverses cardiac and serosal manifestations. This report reinforces the importance of including thyroid function testing in the early evaluation of unexplained effusions and underscores the need for coordinated multidisciplinary management to prevent complications during metabolic recovery.
